# Fees for Life Insurance Examinations

**Published:** 1870-07

**Authors:** 


### Editorial.

### *Fees for Life Insurance Examinations.*

The American Medical Association, at its late meeting passed a resolution fixing Five Dollars as the fee for each examination for life insurance. It would be a pleasant state of affairs if this could become also a fixed fact. But so long as otherwise respectable physicians, who would not venture to crowd out brother practitioners from the care of patients, think themselves wholly justifiable in attempting to displace them as life insurance examiners, we think Companies will not be rapid in advancing the fees.

The scramble for positions of this sort is as funny as a Canada scrub race, and would awaken inextinguishable ridicule and laughter were it generally known.

Prices are nowhere "fixed" by resolution, but solely in accordance with the great laws of supply and demand.

